# Evaluation of a Low-Cost Commercial Actigraph and Its Potential Use in Detecting Cultural Variations in Physical Activity and Sleep

**DOI:** 10.3390/s21113774

**Published:** 2021-05-29

**Authors:** Pavlos Topalidis, Cristina Florea, Esther-Sevil Eigl, Anton Kurapov, Carlos Alberto Beltran Leon, Manuel Schabus

**Affiliations:** 1 Laboratory for Sleep, Cognition & Consciousness Research, Department of Psychology & Centre for Cognitive Neuroscience Salzburg (CCNS), Paris-Lodron University of Salzburg, 5020 Salzburg, Austria; pavlos.topalidis@sbg.ac.at (P.T.); cristina.florea@sbg.ac.at (C.F.); esther-sevil.eigl@sbg.ac.at (E.-S.E.); 2 Department of Experimental and Applied Psychology, Faculty of Psychology, Taras Shevchenko National University of Kyiv, 03680 Kyiv, Ukraine; ankurapov@knu.ua; 3 Department of Clinical Neurophysiology, Institute of Neurology and Neurosurgery, 10200 Havana, Cuba; carlos.beltran@inn.sld.cu

**Keywords:** actigraphy, low-cost fitness trackers, Xiaomi Mi band, ActiGraph, physical activity, sleep, cultural variations, consumer device

## Abstract

The purpose of the present study was to evaluate the performance of a low-cost commercial smartwatch, the Xiaomi Mi Band (MB), in extracting physical activity and sleep-related measures and show its potential use in addressing questions that require large-scale real-time data and/or intercultural data including low-income countries. We evaluated physical activity and sleep-related measures and discussed the potential application of such devices for large-scale step and sleep data acquisition. To that end, we conducted two separate studies. In Study 1, we evaluated the performance of MB by comparing it to the GT3X (ActiGraph, wGT3X-BT), a scientific actigraph used in research, as well as subjective sleep reports. In Study 2, we distributed the MB across four countries (Austria, Germany, Cuba, and Ukraine) and investigated physical activity and sleep among these countries. The results of Study 1 indicated that MB step counts correlated highly with the scientific GT3X device, but did display biases. In addition, the MB-derived wake-up and total-sleep-times showed high agreement with subjective reports, but partly deviated from GT3X predictions. Study 2 revealed similar MB step counts across countries, but significant later wake-up and bedtimes for Ukraine than the other countries. We hope that our studies will stimulate future large-scale sensor-based physical activity and sleep research studies, including various cultures.

## 1. Introduction

In the past three years, the number of wearable devices worldwide has more than doubled, increasing from 325 million in 2016 to 722 million in 2019, while the number of these devices is forecast to reach more than one billion by 2022 [1]. The current trend for health-tracking wearable technologies, such as smartwatches, in combination with the wearables’ software and hardware upgrade capabilities makes their use in scientific inquiries more and more appealing. However, the cost, measurement precision, and, most of all, “black-box” nature of literally all consumer-level devices is a barrier for seriously considering them for answering scientific questions to date. The availability of reliable, accurate, and low-cost commercial smartwatches is expected to rapidly increase their use in scientific endeavors for addressing rest- and activity-related questions in large samples. Following this line of reasoning, we here aimed at (i) directly evaluating the performance of a common and low-cost commercial smartwatch, namely the Xiaomi Mi Band (MB), and (ii) using it for a first cultural study investigating potential cultural differences in physical activity and sleep. We hope that our studies will guide and stimulate future large-scale sensor-based physical activity and sleep research projects using and evaluating similar consumer devices.

Physical inactivity has a negative health effect worldwide [2] and has been related to early age mortality, as well as chronic disease (e.g., breast and colon cancer, diabetes, etc.) [3]. Its promotion has been thus implemented in global health policies [4]. The efficacy of such health policies can be estimated by objectively measuring levels of activity. However, the task of objectively measuring levels of activity is difficult and expensive when applied to large populations and poses some challenges to researches [5]. Although there is no inexpensive “gold standard” for objectively measuring physical activity, body movements measured by wearable accelerometers, such as those found in commercial smartwatches, have been widely used by researchers to capture physical activity data over the course of days and weeks [6,7,8,9]. The number of steps is considered a common objective measure of physical activity among scientific studies: 73.4 precent of the studies investigating physical activity reported step count as a physical activity metric [10].

Next to physical activity, sleep has been also recognized as playing a crucial role in health [11,12,13,14]. The gold standard for measuring sleep is polysomnography (PSG), a combination of electroencephalography (EEG), electrooculography (EOG), electromyography (EMG), and electrocardiography (ECG). Interestingly, commercial smartwatches often also promise to index sleep, based on activity patterns and even heart activity [15]. Many studies consequently have focused on validating actigraphs and commercial smartwatches against PSG. Mantua et al. [16], for example, found no difference between a research-based actigraph and PSG in total-sleep-time and sleep efficiency (see also Lüdtke et al. [17]), but as expected, actigraphy performed poorer on a more finely grained analysis of sleep stages. Similar conclusions have been recently drawn by others who evaluated the performance of commercial smartwatches against PSG, pointing out that although the consumer-grade wearable devices are not yet appropriate for quantifying sleep at high resolution or identifying sleep stages over the night, they still allow adequate estimates of sleep/wake distributions [18,19].

The cost of commercial smartwatches and trackers used in scientific studies ranges from ~EUR 50 to 200 [20], while the scientific devices vastly exceed these costs [21], but also do usually come with various (medical) certifications. Recently, a few studies have drawn attention to low-cost smartwatches by directly comparing their performance in extracting physical activity and sleep measures to that of high-cost actigraphs (including scientific ones) in both laboratory [22,23] and natural conditions [24]. These studies challenge the idea that low-cost actigraphs perform much worse than high-cost commercial actigraphs or even research-based actigraphs in extracting step counts or sleep-related variables.

Such a promising low-cost commercial actigraph (~EUR 30) for measuring physical activity and sleep is the Xiaomi Mi Band (MB) [25,26,27]. Degroote et al. [24] examined in a within-subject three-day free-living protocol the validity of six low-cost devices vs. the GT3X+ actigraph. Among the low-cost actigraphs, the MB achieved—in that study—the highest step correlation agreement with GT3X+ (Spearman’s rho = correlation = 0.91; width limits of agreement: 7450 steps), similar to the performance of the high-cost actigraph (Fitbit: ~EUR 125). However, when the authors compared the MB to a multisensor, which was validated against PSG (SenseWear: BodyMedia Inc., Pittsburgh, PA, USA) in Shin et al. [28], they found that the MB device showed low agreement in total-sleep-time and large deviations. Xie et al. [23] also evaluated the accuracy of MB by comparing it with manually counted steps and sleep-time and found a high MB accuracy for both measures and even higher accuracy than a high-cost commercial smartwatch (Apple Watch). In another study that examined the performance of commercial actigraphs in continuous and intermittent walking, Hartung et al. [29] found that the MB underestimated on average 10/250 steps compared to the actual observed step count in the continuous walking condition, while 63/250 steps in the intermittent walking condition. In both conditions, the MB performed better than all high-cost smartwatches, but worse than the GT3X. The performance of MB to track sleep has been evaluated against PSG in an epoch-by-epoch analysis only in one study so far [30]. Specifically, Ameen et al. [30] found a mean overestimation of the MB device for a total-sleep-time of 69 min. In conclusion, the MB appears to be a promising low-cost smartwatch for measuring physical activity with unclear or yet ambiguous results for sleep-related measures.

An interesting question that could be addressed using consumer device measures on large samples is how cultural variations may influence physical activity and sleep habits across countries. Studying sleep in diverse cultures may allow new insights into human sleep characteristics that can seriously challenge established clinical and scientific views of human sleep [31], as published studies are widely based on WEIRD (westernized, educated, industrialized, rich, and democratic) samples [32]. For example, Yetish et al. [33] investigated sleep in preindustrial societies (e.g., the San in Namibia or the Hadza in Tanzania), using actigraphy-based sleep measures, and found that sleep duration in these societies surprisingly amounted to near the low end of the observed sleep duration in Western industrial societies (see also de La Iglesia et al. [34]). In recent reviews and meta-analyses using objective and subjective sleep measures, Johnson et al. [35] found that racial/ethnic minorities had lower sleep duration and sleep quality than white individuals, while at the same time, Petrov and Lichstein [36] observed that black individuals tend to display greater sleep variability than white individuals. In a large single-day survey in 10 countries, Soldatos et al. [37] found that the average bedtime on weekdays was 11 PM and shifted to 12 PM on weekends. They also observed later bedtimes for Spain and Portugal compared to other countries. More recently, Florea et al. [38] used subjective reports and compared the sleeping trends of a few European countries (Austria, Germany, Ukraine, Greece) and one Caribbean country (Cuba) and observed that Cuba and Ukraine reported longer sleep duration than Austria and Greece, while Ukraine and Greece displayed significantly later bedtimes and wake-up-times than both Cuba and Austria. To our knowledge, there is no study yet that has used actigraphy-derived objective physical activity and sleep measures to directly compare cultural variations across countries.

In the current study, we take advantage of a low-cost actigraph (MB) for tracking objective rest and activity data across countries. First, in Study 1, we aimed to evaluate the performance of MB at extracting reliable movement and sleep-related measures by comparing the MB-based step counts and sleep-times (wake-up-, bed-, and total-sleep-times) to subjective reports and to the GT3X scientific actigraph (wGT3X-BT: ActiGraph, Pensacola, FL, USA). The GT3X is one of the most widely used research actigraphs in extracting physical activity metrics [10,39], but it is more expensive (2.5 times) and heavier (3.87 times) than MB. At the same time, MB is one of the least studied commercial actigraph for both step and sleep research [40]. Having concluded from Study 1 that the MB performed at least equally well as the GT3X, we conducted a second study (Study 2) in which we collected and compared movement and sleep data in Austria, Germany, Cuba, and Ukraine. As this study took place during the COVID-19 pandemic, we opted for these countries ad hoc and where we already had collaboration partners and could compare current data to previously published results from subjective sleep reports (i.e., in Florea et al. [38]).

## 2. Study 1: Evaluation of Mi Band 3 Performance in Tracking Sleep and Physical Activity

### 2.1. Methods and Materials

Participants: For the evaluation of MB performance in extracting movement and sleep measures, the data of 21 healthy participants (females= 13; mean age = 32.1, SD = 8.68) across several days (mean = 8; range: 5–13) were acquired and analyzed. Only participants who did not suffer from any movement impairment and/or sleep disorder were included in Study 1.

Materials: We used the Xiaomi Mi Band 3 (MB; Xiaomi Corp., Beijing, China) as a wrist-worn commercial activity tracker. Small (15.7 × 10.5 × 40.3 mm), light-weight (7.0 g), and rather inexpensive (~50 EUR), MB detects movements via a triaxial accelerometer. MB was connected with the participants’ smartphone via Bluetooth, and a dedicated MB application (MiFit app) displayed the number of steps, wake-up-times, bedtimes, as well as total-sleep-times for each day. Wake-up-times and bedtimes reflected the absolute time that the MB algorithm pinpointed these events, and it was thus measured in time (e.g., hour:minute). In contrast, total-sleep-time was measured in time duration and was calculated as the time interval between bedtime and wake-up-time having excluded periods of detected wakefulness. The experimenter extracted participants’ data from the app at the end of the experiment.

**Figure A1:**
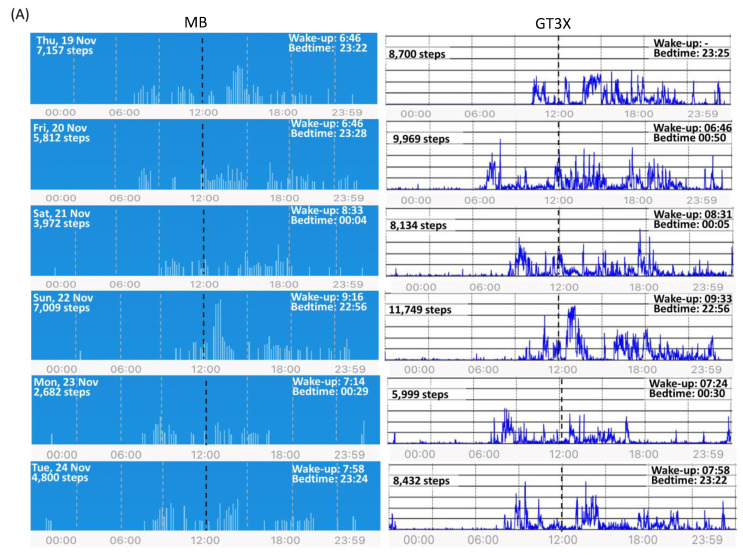

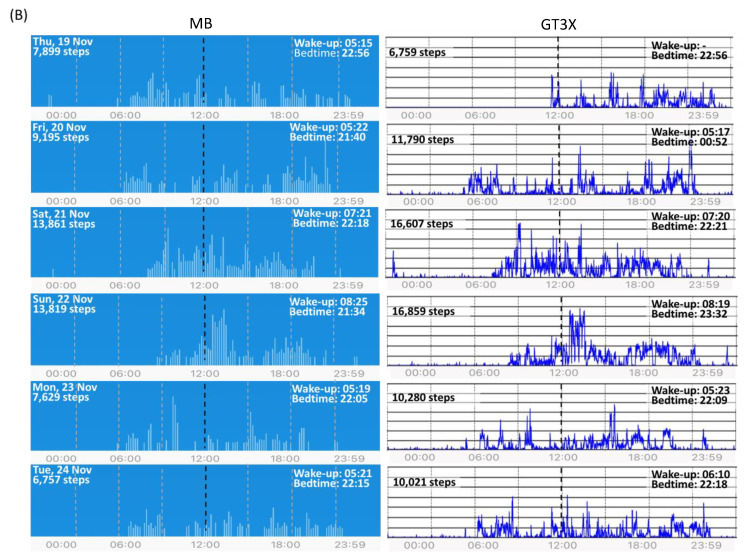
Exemplary step counts as extracted by MB and the GT3X for two participants (**A**,**B**). Note that on many occasions, the GT3X (right) counted steps when MB (left) did not. Note that the GT3X marked some periods as active, whereas for the same periods, MB indicated 0 activity.

In addition, we used the GT3X actigraph (wGT3X-BT: ActiGraph, Pensacola, FL, USA), which is a small (10.5 × 30.3 × 40.6 mm) and light-weight (19 g) commercially available triaxial accelerometer, initially designed for pharmaceutical, healthcare, and research purposes. It measures acceleration in the anteroposterior, lateral, and vertical axes [41] and can be used to index both physical activity levels (i.e., number of steps) and sleep parameters. We used the ActiLife (Version 6.13.1, ActiGraph, Pensacola, FL, USA) data analysis software to extract the variables of interest. To extract the number of steps, we used the default settings of ActiLife for step count: a sampling rate of 30 Hz in a time window of 60 s. For extracting sleep parameters, we opted for the Cole–Kripke [42] sleep algorithm, which is the default option of the ActiLife software. An example of the recordings of the two actigraphs is illustrated in Appendix Figure A1.

Subjective sleep reports were collected using a digital sleep and activity spreadsheet, where people were asked to report on a daily basis their wake-up-times, and bedtimes. Subjective total-sleep-time was calculated post hoc by computing the absolute difference between subjective bedtimes and wake-up-times, and contrary to objective measures, it did not exclude the time that participants spent awake.

Study protocol: On the day of recruitment, participants were instructed to wear the two actigraphs on their non-dominant hand for at least 7 days and to only take them off in the case of showering or swimming, since the GT3X is not water resistant. MB was connected to the participants’ smartphone via a dedicated MB smartphone application that was downloaded on the participants’ phone. In order to not influence participant’s subjective sleep reports, we instructed them not to look at the MB application outputs, but only check on the last day of the experiment, when the data would be extracted. In order to examine whether the relative wrist position of the two actigraphs influenced the results, we randomly assigned participants into one of two groups: one group wore MB closer to the wrist, whereas the other wore the GT3X closer to the wrist. This allowed us to examine the potential effects of the actigraph’s position (i.e., closer vs. further from the wrist) on the estimation of movement and sleep parameters. Eleven out of twenty-one participants wore MB closer to the wrist. Participants were also instructed to fill out a digital sleep log each day, where they reported their daily activity levels, sleep-times, namely wake-up-time, bedtime, and total-sleep-time, as well as their working status (free vs. working day). On the last day of their participation, they returned the actigraphs, and their movement and sleep data were extracted both for MB and the GT3X. The studies were conducted according to the ethical guidelines of the Declaration of Helsinki.

Data and statistical analysis: Firstly, we examined whether the actigraph position relative to the wrist had an effect on the movement and sleep parameters by running a two-sample *t*-test (closer vs. further) for MB and the GT3X separately. We then used a bivariate non-parametric correlation (Spearman’s rho) for indexing the correlation between the two methods for both step and sleep analysis. We investigated to what extent the MB × subjective sleep correlation coefficients differed statistically from the GT3X × subjective sleep correlation coefficients by using the z of Pearson and Filon [43] as implemented in the “cocor” R package [44]. We also used Bland–Altman plots to quantify the methods’ agreement [45,46]. Upper and lower limits of agreement were calculated as the mean difference Âś 1.96 × standard deviations. The limits of agreement are a measure of precision and show the range of values expected for 95% of the observations [47]. We performed the analysis using the “blandr” R package [48]. In addition, we used a simple linear regression to examine whether the biases remained stable with increasing mean values. In order to investigate whether the biases of the two actigraphs differed significantly relative to subjective reports, we compared their biases in reference to the subjective sleep parameters by using a paired-sample *t*-test. All statistical analyses were performed in R, Version 3.6 [49].

### 2.2. Results

#### 2.2.1. Impact of Actigraph Wrist Position on Step Count and Sleep Measures

**Figure A2:**
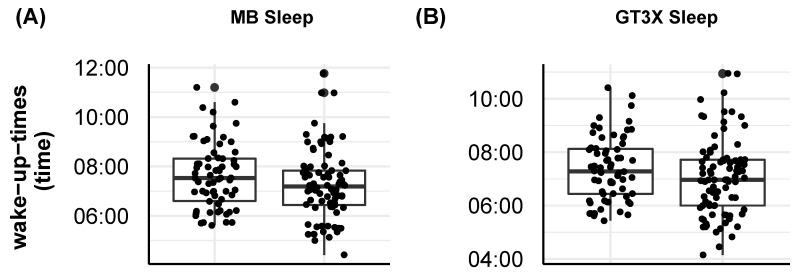

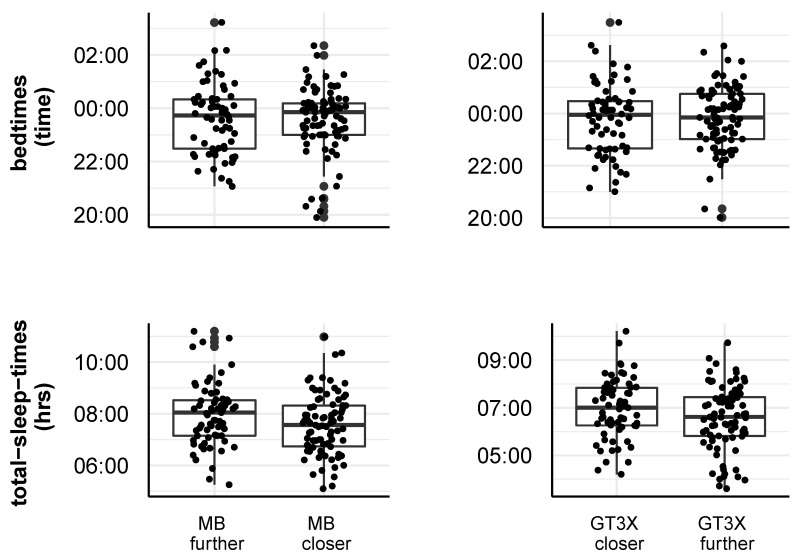
Impact of actigraphy wrist position (further vs. closer to the wrist) on the MB (A) and GT3X (B) sleep measures (top: wake-up-times, middle: bedtimes, bottom: total-sleep-times). The limits of the boxplots represent the 95% CI, while the horizontal middle line represents the median.

The actigraph’s wrist position (further vs. closer to the wrist) did not have an impact on the step counts, not for MB (*t*(134) = 0.97, *p* = 0.33) nor for the GT3X (*t*(135) = −0.09, *p* = 0.93). In addition, actigraph’s position did not influence wake-up-times for either actigraph (MB: *t*(144) = 1.66, *p* = 0.1; GT3X: *t*(149) = 1.55, *p* = 0.123). The same was observed for bedtimes when extracted by MB (*t*(132) = 0.09, *p* = 0.93), as well as the GT3X (*t*(130) = −0.28, *p* = 0.78). However, total-sleep-times were significantly affected for MB (*t*(140) = 2.07, *p* = 0.04), with a closer MB wrist position resulting in shorter total-sleep-times (M = 7.58, SD = 1.17 vs. M = 7.98, SD = 1.21). Interestingly, there was a trend for the GT3X indicating exactly the opposite, namely longer total-sleep-time (*t*(146) = 1.88, *p* = 0.06), with a closer wrist GT3X positioning (M = 6.96, SD = 1.24 vs. M = 6.57, SD = 1.34); see Figure A2.

#### 2.2.2. Agreement between MB and GT3X Step Counts

**Figure 1:**
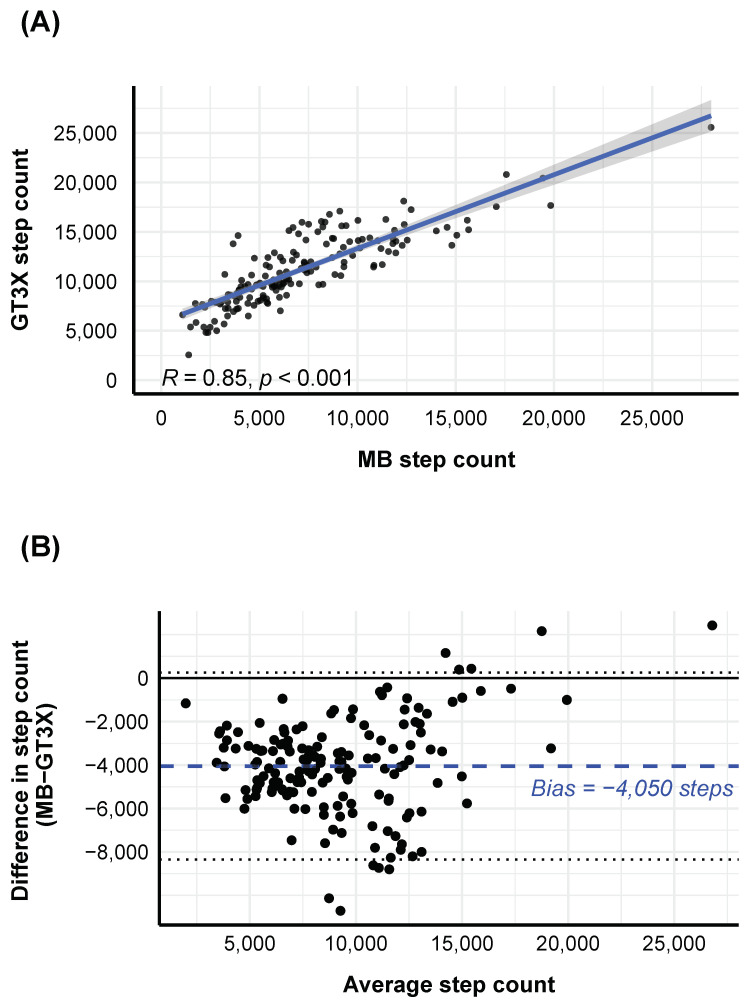
Correlation and agreement analysis between the GT3X and MB on step counts. (**A**) Spearman’s correlation between the GT3X and MB step counts for each day and participant. (**B**) The Bland–Altman plot shows the difference between per-day step count as extracted by the GT3X and MB (y axis) and their average (x axis). Note the high agreement between the two devices (**A**), although there is a clear bias of the MB device to underestimate steps especially in cases with fewer average steps per day (**B**). The dashed blue line illustrates the mean difference (i.e., bias) between the two measurements; the dotted lines represent the 95% CI limits of the mean difference; and the black solid line represents the point of equality (where the difference between the two devices is equal to 0).

As illustrated in Figure 1A, there was a strong positive correlation between the MB and GT3X daily step counts (rho = 0.85, *p* < 0.001). At the subject-level analysis, eighteen out of twenty-one participants had significant and strong correlations (mean rho = 0.88, range: 0.79–0.98) between the MB and GT3X step counts. The degree of agreement between MB and the GT3X was quantified and visualized using a Bland–Altman plot (see Figure 1B). Relative to the GT3X step count, there was an MB step count underestimation, i.e., it estimated on average 4050 (SD = 2195) steps less than the GT3X. In addition, a simple linear regression was calculated to predict the step count difference between the two devices based on the step count mean. We found a significant regression equation (*F*(1,160) = 8.2, *p* < 0.001) with an $R^{2}$ of 0.05, indicating that the difference between the two devices decreased as the average step count increased.

#### 2.2.3. Agreement between Subjective and Actigraphy-Based Sleep Measures

**Figure 2:**
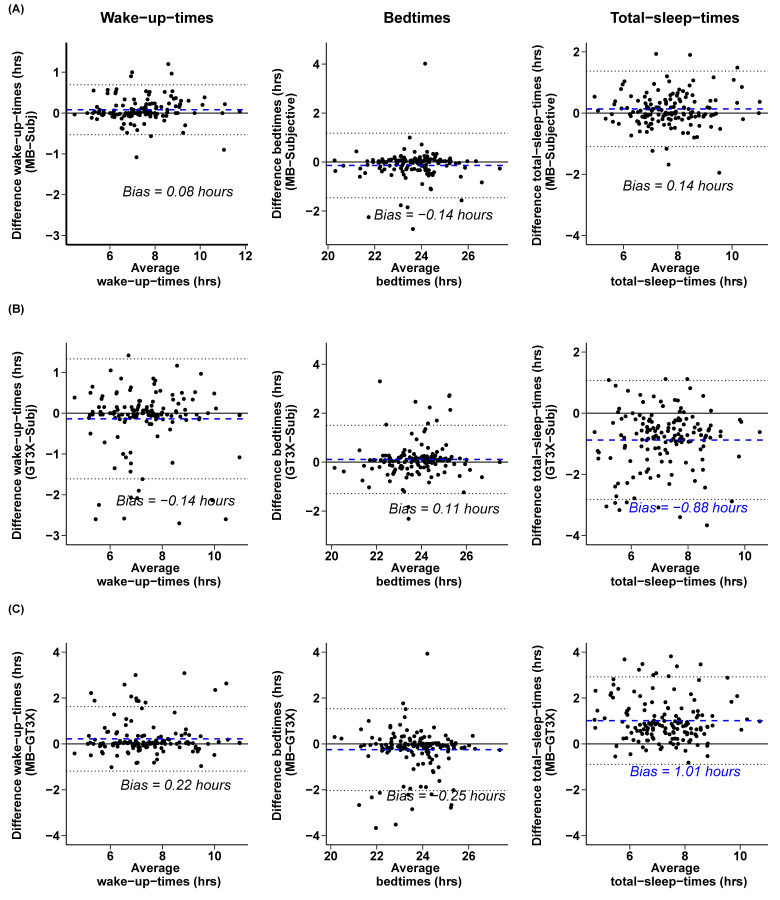
Agreement between the objective and subjective sleep measures: MB vs. subjective (**A**), GT3X vs.subjective (**B**), and the two objective methods MB and GT3X (**C**) as visualized by Bland–Altman plots for wake-up-times, bedtimes, and total-sleep-time. Note the high bias and increased deviations between the GT3X and both subjective and MB total-sleep-times. The dashed lines represent the mean difference (i.e., bias) between the two measurements. The dotted blue lines mark the 95% CI limits of the mean difference, and the black solid line represents the point of equality (where the difference between the two devices is equal to 0).

Subjective sleep reports were also correlated with sleep parameters as extracted by the MB and GT3X algorithms and their agreement was visualized using Bland-Altman plots (cf. Figure 2 for an overview). *Wake-up-times:* The analysis of wake-up-times showed high correlations between subjective and objective parameters for both devices (MB: rho = 0.96, *p* < 0.001; GT3X: rho = 0.84, *p* < 0.001). However, these correlations differed significantly (z = 5.99, *p* < 0.001), indicating statistically higher correlation coefficients between MB and subjective wake-up-times than the GT3X and subjective reports. There was also a significant correlation between the extracted wake-up-times of the two devices (rho = 0.85, *p* < 0.001). The Bland–Altman plot on MB and subjective wake-up-times showed that, on average, MB estimated wake-up-times with a minimal mean bias of M= 0.082 h later than subjectively reported (SD = 0.312 h). *Bedtimes:* The analysis of bedtimes showed high and significant correlations between subjective bedtimes and both objective methods (MB: rho = 0.85, *p* < 0.001; GT3X: rho = 0.83, *p* < 0.001). These correlations did not statistically differ (z = 0.6, *p* = 0.547). The MB bedtimes were also significantly correlated with the GT3X bedtimes (rho = 0.74, *p* < 0.001). The Bland–Altman plot on MB and subjective bedtimes showed that, on average, the MB detected bedtimes 0.14 h earlier than subjective self-reports (SD = 0.67). *Total-sleep-time:* Subjective total-sleep-time correlated significantly with the MB total-sleep-time (rho = 0.86, *p* < 0.001). A lower, but significant correlation was observed between subjective total-sleep-time and GT3X total-sleep-time (rho = 0.73, *p* < 0.001). These correlations differed also significantly (z = 4.47, *p* < 0.001), suggesting a better fit of MB with respect to subjective values. The MB total-sleep-times were also significantly correlated with the GT3X total-sleep-times (rho = 0.72, *p* < 0.001). The Bland–Altman plot on MB and subjective total-sleep-time pointed to an overestimation of 0.14 h (SD = 0.629) by the MB device. The mean difference between MB and subjective total-sleep-time (M = 0.14, SD = 0.63) was statistically different from the GT3X and subjective total-sleep-time differences (M = −0.88, SD = 0.99; *t*(151) = 12.85, *p* < 0.001), indicating that the GT3X underestimated total-sleep-time, whereas MB tended to overestimate total-seep-times as compared to subjective reports (cf. Figure 2).

## 3. Study 2: Cultural Variations in Objective Physical Activity and Sleep

### 3.1. Methods and Materials

Participants: For Study 2, we recruited 124 healthy participants from Austria, Germany, Cuba, and Ukraine with the help of our international collaboration partners. Due to only a small number of German participants (*N* = 8), Austrian and German participants were merged together, as they were considered culturally similar, and are referred as the Austria and Germany group. Only participants with data on both free and workdays were included in this study. Days with less than 1000 steps a day were identified as days where the actigraph was only partially worn and thus excluded from further analysis. The data of 99 participants (females = 60; mean age = 35.34, SD = 8.66, age-range = 19–62) were included in the final analysis. Information about the demographics of each country is displayed in Table 1. In the final sample, a mean of 10.69 days (range: 7–47) per participant was recorded and analyzed.

Materials: In Study 2, we used exclusively the MB device that was evaluated in Study 1. The MB actigraphs were distributed to each country, and collaboration partners used them to extract objective movement and sleep parameters. In addition, subjective movement and sleep measures were obtained via daily logs in the online survey platform LimeSurvey [50].

Study protocol: On the day of recruitment, participants received the MB actigraph and were instructed to wear it for 7 consecutive days and at least 2 free days on their non-dominant hand, then go on with their everyday activities as usual. Having observed a wrist position effect on a variable of interest in Study 1, participants were given precise instructions regarding the position and the tightness of the MB actigraph: “1 cm above the wrist, a comfortable level of tightness, not too tight and not too loose”. For each day of participation, participants were asked to fill out an online activity and sleep log. The study was conducted according to the guidelines of the Declaration of Helsinki and approved by the Institutional Ethics Committee of the University of Salzburg.

Data and statistical analysis: In order to avoid data from days on which the actigraphs were partially worn, the data from the first day (day of recruitment) for every participant were discarded. The data from free and workdays were averaged, resulting in a single value per participant, for all objective and subjective physical activity and sleep variables. This split between work and free days reflected differences in sleep characteristics on those days (e.g., Pilz et al. [51]). In addition, we applied a 3-SD outlier filter on sleep data, to filter out days with extreme values. Due to a significant country effect on age (*F*(2, 195) = 11.859, *p* < 0.001, $\eta_{p}^{2}$ = 0.108), we investigated the main effects and interactions while controlling for age. Thus, we performed a 2 × 3 analysis of covariance (ANCOVA) with day (free vs. work) as a within-subject factor and country (Austria/Germany vs. Cuba vs. Ukraine) as a between-subject factor and age as a covariate. We followed up significant main effects and interaction using Bonferroni-corrected pairwise marginal mean comparisons, and thus, we reported only the comparisons that reached a significance threshold of *p* < 0.01. As Study 2 took place partly during the COVID-19 pandemic, we examined also if there was any effect of the lockdown status on step counts. The participants were characterized as “in” or “out of” lockdown based on the official lockdown dates of each country. Statistical analysis was performed in R, Version 3.6 [49].

### 3.2. Results

#### 3.2.1. Subjective Physical Activity and Objective Step Count Differences across Countries

**Figure 3:**
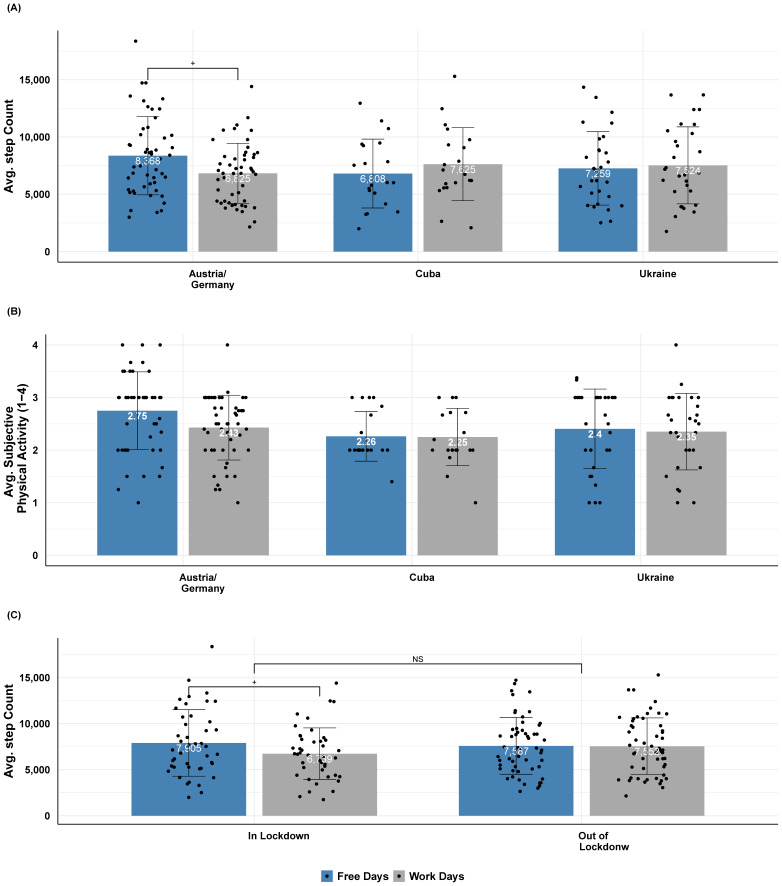
Subjective physical activity and objective step count differences across countries. (**A**) Mean step count for each country (Austria and Germany, Cuba, and Ukraine) and day (work or free), as extracted by MB. Note that it was indicative that in Austria there were more steps during free compared to workdays. (**B**) Mean subjective physical activity (1 = not at all, 4 = a lot) for each country and day. (**C**) Mean MB step count for in and out of lockdown participants, separately for work and free days. Note that there was no significant effect between in and out of lockdown. Error bars indicate the standard deviation of the mean. Bonferroni-corrected pairwise marginal mean comparisons indicate significance at <0.1+, <0.05 *, <0.01 **, <0.001 ***, and non-significant (NS) levels.

We performed an ANCOVA with day (work vs. free) as the within factor and country (Austria/ Germany vs. Cuba vs. Ukraine) as the between factor on MB step count, while controlling for age. There was neither a main effect of country (*F*(2, 95) = 0.27, *p* = 0.76, $\eta_{p}^{2}$ = 0.006), nor a main effect of day (*F*(1, 95) = 0.16, *p* = 0.69, $\eta_{p}^{2}$ = 0). However, there was a significant country × day interaction (*F*(2, 95) = 4.64, *p* = 0.012, $\eta_{p}^{2}$ = 0.089). Post hoc Bonferroni-corrected pairwise contrasts showed a trend for higher step counts on free (M = 8368, SD = 3418) than workdays (M = 6825, SD = 2627) in the Austrian sample, but this was not the case for Cuba and Ukraine (cf. Figure 3A).

Similar results were observed when analyzing subjective physical activity (see Figure 3B). Statistically, there was neither a main effect of country (*F*(2, 95) = 2.74, *p* = 0.07, $\eta_{p}^{2}$ = 0.06), nor a main effect of day (*F*(1, 95) = 0.04, *p* = 0.85, $\eta_{p}^{2}$ = 0), indicating a stable average activity level among countries and between free and workdays. The country × day interaction (*F*(2, 95) = 2.22, *p* = 0.11, $\eta_{p}^{2}$ = 0.045) indicated the same trend as mentioned above, but it was not significant.

Lastly, we examined the effect of the lockdown on step counts and performed a between-subject two-way mixed ANCOVA with day (work vs. free) as a within-subject factor and lockdown status (in vs. out) as a between-subject factor. Interestingly, there was neither a main effect of lockdown status (*F*(1, 96) = 0.2, *p* = 0.66, $\eta_{p}^{2}$ = 0.002), nor day (*F*(1, 96) = 2.62, *p* = 0.11, $\eta_{p}^{2}$ = 0.027), and the day × lockdown status interaction was only marginally significant (*F*(1, 96) = 2.95, *p* = 0.09, $\eta_{p}^{2}$ = 0.03), indicating that “in” lockdown participants tended to move less during work-days (M = 6739, SD = 2799) than free days (M = 7905, SD = 3611) - cf. Figure 3C.

#### 3.2.2. Subjective and Objective Sleep Measure Differences across Countries

**Figure 4:**
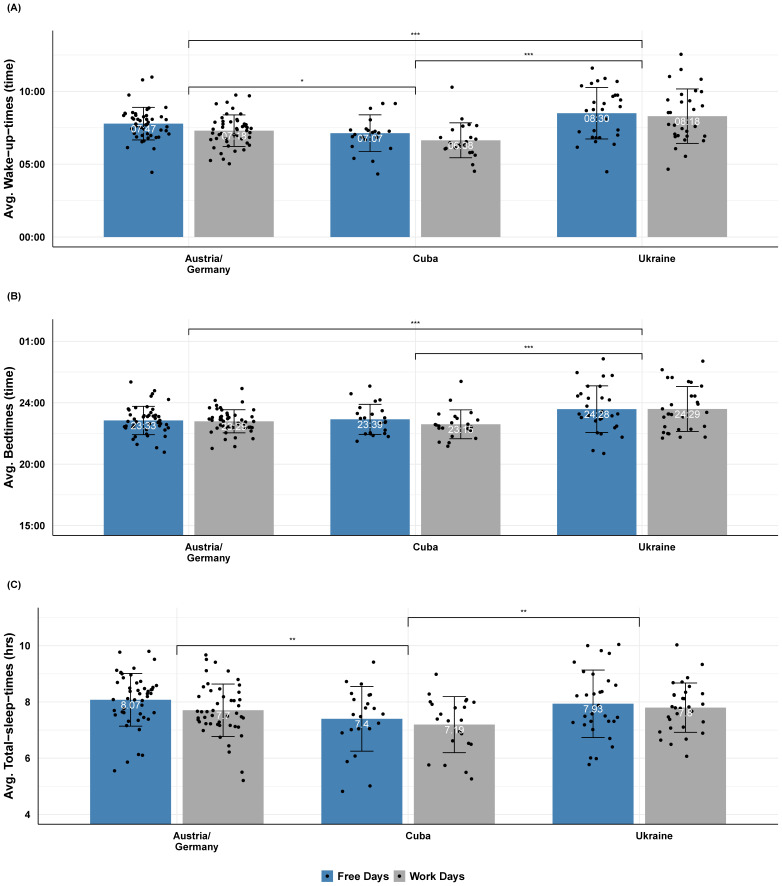
Objective sleep measures across countries. Average wake-up-times (**A**), bedtimes (**B**), and total-sleep-times (**C**) for each country (Austria and Germany, Cuba, and Ukraine) and day (work or free), as extracted by MB. Note that Ukraine had later wake-up and bedtimes than both Austria and Cuba. Austria had later wake-up-times and higher total-sleep-times than Cuba. Error bars indicate the standard deviation of the mean. Bonferroni-corrected pairwise marginal mean comparisons indicate significance at <0.1 +, <0.05 *, <0.01 **, <0.001 ***, and non-significant (NS) levels.

We performed a 2 × 3 mixed ANCOVA with day (work vs. free) as a within-subject factor and country (Austria/Germany vs. Cuba vs. Ukraine) as a between-subject factor on objective sleep measures, as extracted by MB, while controlling for age. *Wake-up-times:* The analysis of wake-up-times showed a main effect of country (*F*(2, 91) = 8.1, *p* < 0.001, $\eta_{p}^{2}$ = 0.151), with Ukraine (M = 08:24, SD = 01:48) having significantly later wake-up-times than both Austria (M = 07:33, SD = 01:07) and Cuba (M = 06:53, SD = 01:14). The main effect of day (*F*(1, 91) = 0, *p* = 0.98, $\eta_{p}^{2}$ = 0), as well as the country × day interaction (*F*(2, 91) = 1.34, *p* = 0.27, $\eta_{p}^{2}$ = 0.029) were not significant (cf. Figure 4A). Similar results were also observed when analyzing subjective wake-up-times: there was a significant main effect of country (*F*(2, 93) = 7.42, *p* < 0.001, $\eta_{p}^{2}$ = 0.138), but the main effect of day (*F*(1, 93) = 0.27, *p* = 0.6, $\eta_{p}^{2}$ = 0.003) and the country × day interaction (*F*(2, 93) = 0.99, *p* = 0.38, $\eta_{p}^{2}$ = 0.021) were not significant. *Bedtimes:* The ANCOVA on MB bedtimes showed a significant main effect of country (*F*(2, 91) = 7.94, *p* < 0.001, $\eta_{p}^{2}$ = 0.149): Ukraine (M = 24:29, SD = 01:51) had significantly later bedtimes than both Austria (M = 23:27, SD = 01:12) and Cuba (M = 23:32, SD = 01:03). The main effect of day (*F*(1, 91) = 0.65, *p* = 0.42, $\eta_{p}^{2}$ = 0.007), as well as the country × day interaction (*F*(2, 91) = 0.75, *p* = 0.48, $\eta_{p}^{2}$ = 0.016) were not significant (cf. Figure 4B). Likewise, analysis of subjective bedtimes showed a significant main effect of country (*F*(2, 91) = 7.47, *p* < 0.001, $\eta_{p}^{2}$ = 0.141), while the main effect of day (*F*(1, 91) = 0.26, *p* = 0.61, $\eta_{p}^{2}$ = 0.003), as well as the country × day interaction (*F*(2, 91) = 0.12, *p* = 0.88, $\eta_{p}^{2}$ = 0.003) were not significant. *Total-sleep-time*: The ANCOVA on the MB total-sleep-times showed a trend for a main effect of country (*F*(2, 93) = 3, *p* = 0.06, $\eta_{p}^{2}$ = 0.061): both Austria (M = 07:53, SD = 00:57) and Ukraine (M = 07:52, SD = 01:03) had higher total-sleep-times than Cuba (M = 07:18, SD = 01:04). The main effect of day (*F*(1, 93) = 0.03, p = 0.86, $\eta_{p}^{2}$ = 0), as well as the country × day interaction (*F*(2, 93) = 0.54, *p* = 0.58, $\eta_{p}^{2}$ = 0.012) were not significant (cf. Figure 4C). We observed similar effects when performing the same analysis on subjective total-sleep-times (country: *F*(2, 93) = 2.47, p = 0.09, $\eta_{p}^{2}$ = 0.05); day: (*F*(1, 93) = 0.29, *p* = 0.59, $\eta_{p}^{2}$ < 0.001; country × day: *F*(2, 93) = 0.4, p = 0.67, $\eta_{p}^{2}$ = 0.009).

## 4. Discussion

In Study 1, we evaluated the performance of MB to objectively measure physical activity and sleep measures. The low-cost of MB in combination with its promising performance in extracting physical activity and sleep-related measures [23,24,29,30] endorsed the utilization of this device for a cross-cultural actigraphy study. We revealed that step counts were not influenced by the exact actigraph wrist position, neither for MB, nor for the GT3X. However, the MB device appeared less sensitive to body movements after sleep onset when placed further away from the wrist, likely resulting in decreased detection of fragmented sleep and, consequently, longer detected total-sleep-times.

Furthermore, we revealed that the MB step counts were strongly correlated to the scientific GT3X device, but with an obvious underestimation of steps especially in days with few steps. Thus, our results indicated that although MB captured the fluctuations in movement patterns similarly well to the scientific GT3X device (see also Appendix Figure A1), when looking at raw absolute values, there were strong differences. Our results were widely in line with those reported in Degroote et al. [24], showing a high MB ~GT3X+ correaltion (r = 0.91). However, Degroote et al. [24] found weaker MB vs. GT3X underestimation (i.e., 1000 steps) compared the one found in the current study (i.e., 4000 steps). Part of this disagreement could be expailned by including only 44 days of recorded data under free-living conditions in a sample of 20 participants in Degroote’s study, compared to 162 days and 21 participants analyzed in the current study (mean = 8; range: 5–13). According to Hartung et al. [29], MB counted 63 out of 250 manually counted steps too few, whereas the GT3X counted even 30 steps more in a natural walking condition, confirming the general underestimation of the MB device for step counts. In a recent study, Höchsmann et al. [52] found that the wrist GT3X+ step count was overestimating steps especially during low, but active (e.g., public transport or car driving) or even inactive (e.g., sitting or laying) situations, when compared with ankle-based measurements (see also Figure A1). Thus, in principle, the differences between the MB and GT3X step counts in the current study could also be explained by an overestimation of the GT3X wrist step count in free-living conditions, which is also in line with the observation of Hartung et al. [29]. Based on the current results, we therefore concluded that the MB adequately captured activity fluctuations across days, but raw absolute values were hard to compare and differed widely between actigraphy devices.

Furthermore, all the MB sleep measures were strongly correlated with subjective measures (Spearman’s rho > 0.85) and appeared even more accurate than the GT3X estimations (using the standard settings), e.g., in wake-up- and total-sleep-time estimations. The underestimation of the GT3X total-sleep-times compared to subjective reports (Figure 2) could be explained by taking under consideration that the subjective total-sleep-times did not include potential awake time after sleep onset. In addition, the GT3X appeared to be sensitive to body movements during sleep that might have resulted in calculating fragmented sleep and thus smaller total-sleep-times (also see Figure A1). It is important to note that although the participants were instructed to not look at their smartphone application where the MB sleep data could be displayed, we had no means of validating that some subjects did not check their MB data on some days before filling in their subjective sleep reports. However, the results from Xie et al. [23], who found high accuracy for the MB sleep duration when compared to manual sleep timing, are reassuring. In addition, Kubala et al. [53] also found an overestimation of the MB total-sleep-time (in the range of 53–80 min) when compared to a research-based actigraph (ActiWatch: Philips Respironics, Murrysville, PA, USA). Furthermore, we considered the use of subjective sleep reports for evaluating the performance of MB in extracting sleep-related variables as a limitation of the current study, but this was an ad hoc study during the COVID-19 pandemic that made the use of PSG impossible. Future studies with a focus on assessing the performance of low-cost actigraphs in extracting sleep parameters should include PSG as the “gold-standard”, as has been previously done [30,54]. In the current study, we used the Xiaomi Mi Band Model 3, which to our knowledge has no major hardware/sensor changes compared to the Xiaomi Band Model 2, so discrepancies between the studies are unlikely to be explained by differences in the hardware of the Xiaomi models [30]. However, since the MB software updates as it advances to the next model, it is possible that some of the above-mentioned discrepancies between the current and previous studies were due to software upgrades.

In Study 2, we investigated whether there were systematic differences among Austria and Germany (grouped as they were considered culturally similar), Cuba, and Ukraine in physical activity and sleep habits. Our results showed that daily physical activity as indexed via the MB number of steps did not significantly differ among the three countries, nor between work and free days. The only trend observed in the data was a higher step count in Austria on free days as compared to workdays, which may be explained by more outdoor sports activities during free days. Interestingly, we also observed a trend of less steps “during” COVID-19 lockdown working days as compared to free days or times out of lockdown. This effect across cultures was likely driven by the vastly increased shift to home offices and the closure of bars, restaurants, (indoor) sports activities, and the like and is well in agreement with world-wide survey studies indicating a decrease in physical activity during the pandemic [55].

We also analyzed objective sleep measures as extracted by MB and found that Ukraine obtained significantly later wake-up and bedtimes than Austria and Germany and Cuba. This was in line with previous reports of our work group [38]. However, in contrast to Florea et al. [38], we found a trend for longer total-sleep-time for the Austrian and German than the Cuban sample. In addition, we observed convergent results between objective and subjective analysis for all sleep measures, which speaks to the acceptable approximation of sleep measures of interest with the MB consumer device. It is also worth mentioning that the sample size in the current actigraphy-based study was much smaller than the one used in Florea et al. [38], who focused on survey-based cultural differences. Furthermore, despite the minimum threshold of seven actigraphy days per subject that we set for the current study, the data on free days were still limited compared to the amount of data for workdays. This may have resulted in less precise measurement of the habitual physical activity and sleep on free/leisure days. Future studies should strive to increase the sample sizes and track subjects over longer time periods in order to capture day-by-day and cultural variations more precisely. It is important here to note that although we did not find a significant difference between the step counts of in- and out-of-lockdown participants, the results need to be interpreted with caution since there might be factors that influence cultural variations in both sleep and activity that we did not control for (e.g., system job relevance). However, given that in our earlier study, we did not find cultural differences when it came to sleep changes due to the Coronavirus pandemic, we believe that it was a negligible effect [38]).

Population-level sensor-based studies are needed to better understand how cultural factors or also seasonal changes affect activity levels, as well as rest–wake cycles. Studies purely relying on subjective measures are a good starting point [56], but of course suffer from problems such as recall bias, inherent problems of estimating sleep onset, sleep-time or nightly awakenings for states that are known to be “unconscious”, or simple socially desired response bias. These problems can be surpassed with objective tracking of sleep and activity using affordable smartwatches. These devices also have the advantage that they can be used in low-income and developing countries and add to our knowledge of intercultural differences or similarities in sleep habits and activity patterns [18]. Yet, we emphasize that it is inevitable that such low-cost consumer devices are continuously monitored and evaluated by independent research in order to ensure reliable measurements and data, as well as identify limitations.

## Figures and Tables

**Table 1:** Sample’s demographic distrbution of age and gender.

Country	N	Males	Femeales	Age Mean	Age AD
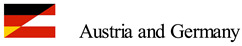	50	20	30	32.8	8.66
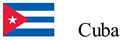	20	10	10	41.1	8.41
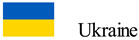	29	9	20	35.34	10.28
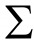	99	39	60	35.34	8.66

## Data Availability

The data presented in this study are available on request from the corresponding author.

## Abbreviations

The following abbreviations are used in this manuscript:

MB	Xiaomi Mi Band
GT3X	wGT3X-BT ActiGraph
